# Radiomics-based prediction of FIGO grade for placenta accreta spectrum

**DOI:** 10.1186/s41747-023-00369-2

**Published:** 2023-09-20

**Authors:** Helena C. Bartels, Jim O’Doherty, Eric Wolsztynski, David P. Brophy, Roisin MacDermott, David Atallah, Souha Saliba, Constance Young, Paul Downey, Jennifer Donnelly, Tony Geoghegan, Donal J. Brennan, Kathleen M. Curran

**Affiliations:** 1 Department of UCD Obstetrics and Gynaecology, School of Medicine, University College Dublin, National Maternity Hospital, Holles Street, Dublin 2, Ireland; 2 Siemens Medical Solutions, Malvern, PA USA; 3 Department of Radiology & Radiological Science, Medical University of South Carolina, Charleston, SC USA; 4 Radiography & Diagnostic Imaging, University College Dublin, Dublin, Ireland; 5 Statistics Department, University College Cork, Cork, Ireland; 6 Insight Centre for Data Analytics, Dublin, Ireland; 7 Department of Radiology, St. Vincent’s University Hospital, Dublin, Ireland; 8 Department of Gynecology and Obstetrics, Hôtel-Dieu de France University Hospital, Saint Joseph University, Beirut, Lebanon; 9 Department of Radiology: Fetal and Placental Imaging, Hôtel-Dieu de France University Hospital, Saint Joseph University, Beirut, Lebanon; 10 Department of Histopathology, National Maternity Hospital, Dublin, Ireland; 11 Department of Obstetrics and Gynaecology, Rotunda Hospital, Dublin, Ireland; 12 Department of Radiology, Mater Misericordiae University Hospital, Dublin, Ireland; 13 University College Dublin Gynaecological Oncology Group (UCD-GOG), Mater Misericordiae University Hospital and St Vincent’s University Hospital, Dublin, Ireland; 14 Systems Biology Ireland, School of Medicine, University College Dublin, Dublin, Ireland; 15 School of Medicine, University College Dublin, Dublin, Ireland

**Keywords:** Machine learning, Magnetic resonance imaging, Placenta accreta, Pregnancy, Radiomics

## Abstract

**Background:**

Placenta accreta spectrum (PAS) is a rare, life-threatening complication of pregnancy. Predicting PAS severity is critical to individualise care planning for the birth. We aim to explore whether radiomic analysis of T2-weighted magnetic resonance imaging (MRI) can predict severe cases by distinguishing between histopathological subtypes antenatally.

**Methods:**

This was a bi-centre retrospective analysis of a prospective cohort study conducted between 2018 and 2022. Women who underwent MRI during pregnancy and had histological confirmation of PAS were included. Radiomic features were extracted from T2-weighted images. Univariate regression and multivariate analyses were performed to build predictive models to differentiate between non-invasive (International Federation of Gynecology and Obstetrics [FIGO] grade 1 or 2) and invasive (FIGO grade 3) PAS using R software. Prediction performance was assessed based on several metrics including sensitivity, specificity, accuracy and area under the curve (AUC) at receiver operating characteristic analysis.

**Results:**

Forty-one women met the inclusion criteria. At univariate analysis, 0.64 sensitivity (95% confidence interval [CI] 0.0−1.00), specificity 0.93 (0.38−1.0), 0.58 accuracy (0.37−0.78) and 0.77 AUC (0.56−.097) was achieved for predicting severe FIGO grade 3 PAS. Using a multivariate approach, a support vector machine model yielded 0.30 sensitivity (95% CI 0.18−1.0]), 0.74 specificity (0.38−1.00), 0.58 accuracy (0.40−0.82), and 0.53 AUC (0.40−0.85).

**Conclusion:**

Our results demonstrate a predictive potential of this machine learning pipeline for classifying severe PAS cases.

**Relevance statement:**

This study demonstrates the potential use of radiomics from MR images to identify severe cases of placenta accreta spectrum antenatally.

**Key points:**

• Identifying severe cases of placenta accreta spectrum from imaging is challenging.

• We present a methodological approach for radiomics-based prediction of placenta accreta.

• We report certain radiomic features are able to predict severe PAS subtypes.

• Identifying severe PAS subtypes ensures safe and individualised care planning for birth.

**Graphical Abstract:**

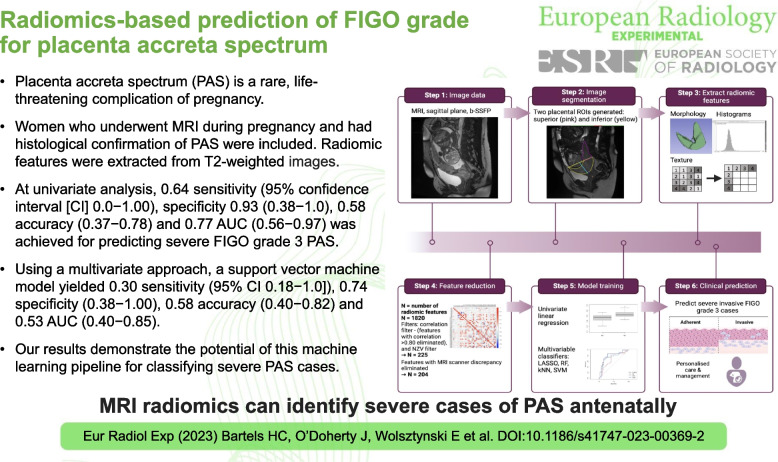

**Supplementary Information:**

The online version contains supplementary material available at 10.1186/s41747-023-00369-2.

## Background

Placenta accreta spectrum (PAS) is a rare, life-threatening complication of pregnancy where the placenta is abnormally attached to the uterine wall [1]. PAS is classified into three grades by the International Federation of Gynecology and Obstetrics (FIGO) [2], with FIGO grade 3 being the most severe. Up to 50% of cases worldwide are undiagnosed during pregnancy, which is associated with significantly poorer maternal and fetal outcomes [3]. Predicting disease severity antenatally remains a major challenge. There is no consensus internationally on optimal management, with variations in many aspects of clinical care [4].

Ultrasound and magnetic resonance imaging (MRI) are the imaging modalities of choice for diagnosing PAS antenatally [5, 6]. While PAS is usually first suspected from an ultrasound assessment, MRI plays an important role, such as for surgical planning and in the assessment of posterior or lateral defects [5, 7, 8].  Furthermore, some centres routinely perform MRI when ultrasound signs of PAS are seen [9]. However, the diagnostic accuracy of MRI is highly dependent on reader expertise and can incorrectly classify cases in up to 30% of cases [10].

Radiomics is a quantitative approach to medical imaging, where potential image biomarkers are extracted from images [11]. Several studies have applied radiomics to MR images in PAS and found radiomic features were useful in aiding diagnosis and predicting clinical outcomes such as massive obstetric haemorrhage [12, 13]. These were summarised in a recent systematic review, which included 10 studies [14]. The review highlighted the varying methodological quality of the radiomics PAS studies to date [14]. Many were limited by the significant heterogeneity in how PAS was defined, with only two studies reporting on histological data, which is considered the reference standard in diagnosing PAS [14, 15]. In one of the largest studies included, over 70% of PAS cases had neither of the two most important and frequent predisposing risk factors [16], which are a prior Caesarean section and placenta previa [17]. Hence it is unclear what clinical or histopathological criteria were used to define PAS in many of these studies [14]. Furthermore, a lack of standardised methodology is an important limitation of all radiomics studies and remains a major challenge in the field [18].

Therefore, the current literature is limited by the lack of standardised definitions for PAS and the varying quality of the radiomic methodology. We propose a methodological approach for image segmentation and a radiomic workflow for predicting severe FIGO grade 3 PAS. We describe the location and severity of PAS using standardised definitions as they are currently understood [2, 15], use a standardised methodology for radiomic feature extraction [19], and provide an open source code for each step, with adherence to the Radiomics Quality Score [20] as much as was feasible. We report our results from a pilot test dataset.

## Methods

### Study population

**Fig. 1:**
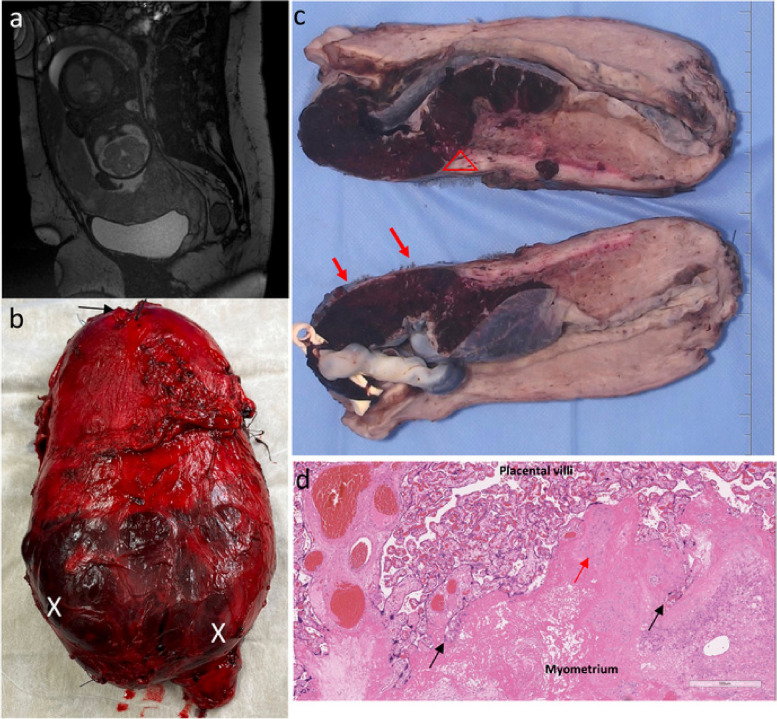
A case of PAS FIGO grade 3: correlation between MRI and histopathology. **a** MRI sagittal view obtained at 30 weeks gestation. Complete placenta previa demonstrating features of PAS including abnormal intraplacental vascularity, myometrial thinning and placental bulge towards the bladder. **b** Fresh hysterectomy specimen showing lower uterine segment bulging and distention with minimal overlying serosa (X) from abnormal placentation. Placenta can be seen through the very thin remaining serosa (X). Arrow marks fundal uterine incision where the baby was delivered. **c** Gross cross section of cut specimen: FIGO 3a with outer 25% of the myometrium involved. Triangle marks area where area of placental "invasion" led to scar dehiscence, with only a thin area of residual myometrium remaining (red arrows). No invasion beyond serosa or involvement of other organs. **d** Microscopy shows invaded placenta with absent decidua basalis, trophoblast cells invading deep into the myometrium (black arrows) as a result of abnormal uterine remodelling from a previous Caesarean scar, and loss of the normal uterine contour. Evidence of chronic inflammation (red arrow) and edema are also present in the myometrium. *MRI* Magnetic resonance imaging, *FIGO* International Federation of Gynecology and Obstetrics, *PAS* Placenta accreta spectrum

Ethical approval was obtained from the National Maternity Hospital, Dublin (EC30.2018) and Rotunda Hospital Dublin ethics committees (RAG 2019–10). Participants provided written, informed consent. Image data was obtained prospectively, and retrospectively analysed as part of a two-centre cohort from between January 2018 to October 2022. Inclusion criteria were: consecutive participants who underwent MRI for suspicion of PAS based on ultrasound assessment [6], intraoperative findings at the time of laparotomy found clinical features of PAS as defined by the FIGO classification [2], and examination by a specialist perinatal histopathologist (> 10 years of experience) who confirmed PAS on histology [15]. Figure 1 shows an example of an included PAS case on MRI, with corresponding gross and microscopic histopathology images. We excluded cases with MRI performed for suspicion of PAS, without clinical evidence of PAS intraoperatively or histopathological examination confirming the diagnosis, or those who gave birth outside of a participating centre. Placenta previa was defined as the placenta completely covering the internal cervical os on transvaginal ultrasound beyond 20 weeks of gestation [21].

### PAS multidisciplinary team management

All participants in this study were cared for by a multidisciplinary PAS specialist team. Antenatal imaging consists of ultrasound, performed by fetal-medicine specialists using both transabdominal and transvaginal ultrasound, and MRI, read and reported by specialist radiologists [22] (T.G., with over 15 years of experience; D.B., with over 20 years of experience). Timing of elective delivery was between week 34 and week 36 of gestation, following a standardised surgical approach [23]. Cases confirmed intraoperatively as PAS undergo either myometrial resection or Caesarean hysterectomy.

### MRI protocol

Patients were scanned on a 1.5-T scanner (Optima 450W MR, General Electric Healthcare, Waukesha, USA) (*n* = 40) or a 1.5-T scanner (MAGNETOM Sola, Siemens Healthineers, Erlangen, Germany) (*n* = 7) using a T2-weighted sagittal two-dimensional balanced steady-state free precession, b-SSFP, sequence with a slice thickness of 4 mm, slice spacing of 1 mm, and a field of view of 38 cm at both imaging sites. The MRI acquisition protocol of the placenta for the assessment of PAS performed at site 1 was previously described [24, 25].

### Radiomics processing

**Fig. 2:**
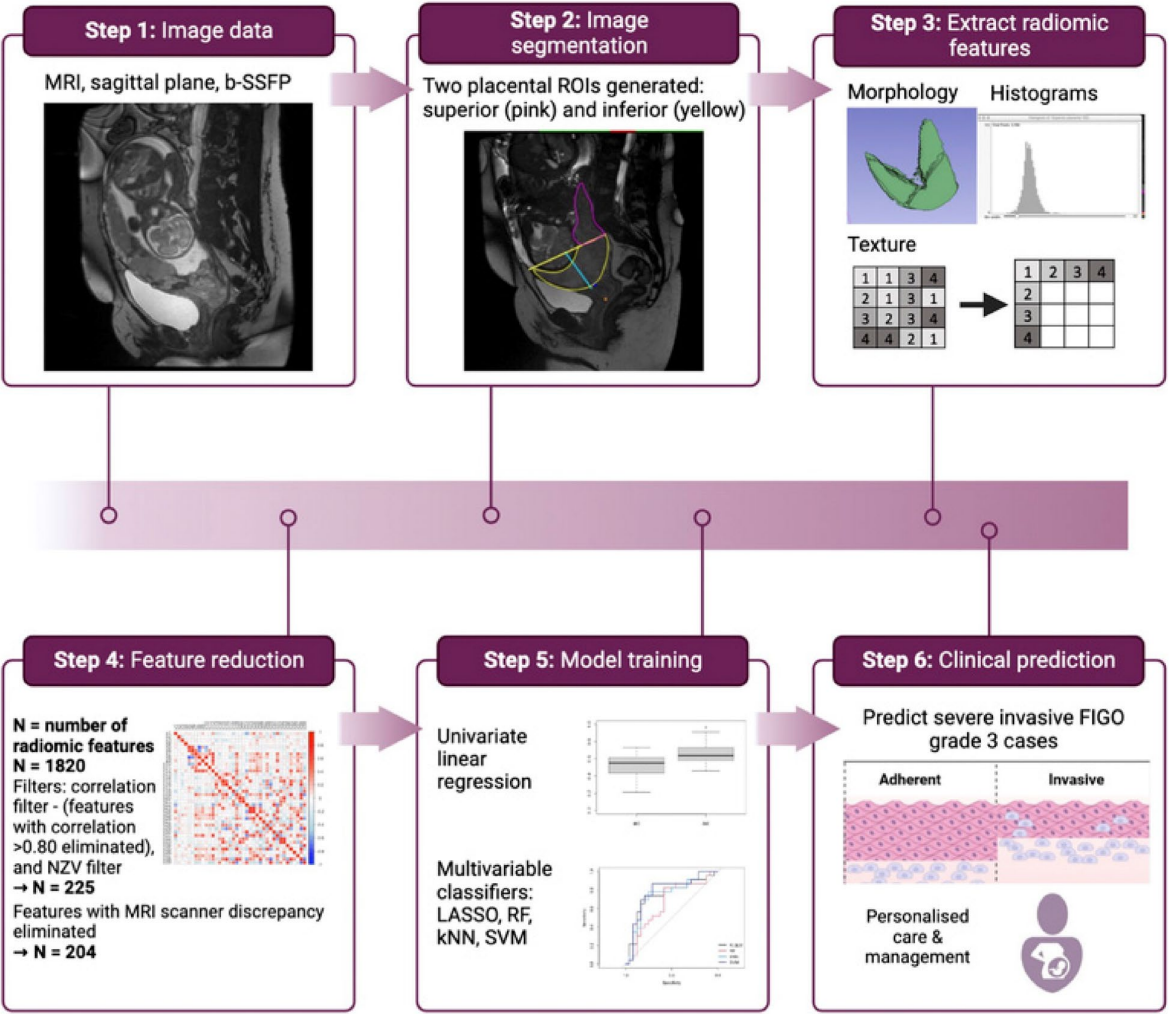
Summary of radiomics processing, feature reduction, and modelling. The N reported throughout applies to the number of radiomic features extracted when using convolutional image filters. *kNN* k-nearest neighbour, *LASSO* Least absolute shrinkage and selection operator, *NZR* Near zero variance, *RF* Random forest, *SVM* Support vector machine

The workflow for radiomics processing is summarised in Fig. 2. The code for these steps and methodology used is publicly available in the following repository: https://github.com/helenabartels91/PASRadiomics.git

### Image segmentation

Sagittal planes were used for segmentation as this allows an optimal view of the bladder, placental location and the cervix, and the relationship of these key organs of interest to each other, including the area of placental adherence or invasion. Images were manually segmented on multiple representative slices with regions of interest (ROIs) by three independent investigators (H.B., D.B., R.M., with 4 over 20 and 6 years of experience). Investigators were aware of the purpose of the study but were blinded to the results of other imaging, or final intraoperative or histopathological outcomes. The segmentation protocol was informed by a previous work where ROIs of the placenta proximal and remote to the prior Caesarean section scar showed significant differences in the distribution of radiomic features, which were associated with undergoing caesarean hysterectomy for PAS [13]. Furthermore, we have previously reported a linear relationship between the distance from the internal cervical os to the most proximal part of the PAS defect as seen on MRI and estimated blood loss, with defects closer to the internal os associated with significantly higher blood loss [26]. Hence, we generated two placental ROIs, based on proximity to the area of adherence or invasive (inferior placental ROI, close to internal cervical os) and remote from this area (superior placental ROI). The image segmentation protocol is included in Supplementary materials.

### Feature extraction

Radiomic feature extraction was performed using PyRadiomics [11], resulting in 106 radiomic features from 6 feature families including shape, first-order (histogram-based) and second-order (Gray Level Cooccurrence Matrix, Gray Level Run Length Matrix, Gray Level Size Zone Matrix, and Gray Level Dependence Matrix) features, from the original image. Feature families and specific features used in this work are mathematically defined as per the PyRadiomics framework, as previously described (https://pyradiomics.readthedocs.io/en/latest/features.html). Separately, feature extraction was performed using convolutional image filters such as Laplacian of Gaussian (with 5 sigma levels: 1 level of wavelet decompositions resulting in eight derived images and images derived using square, square root, logarithm and exponential filters) resulting in another 1,714 radiomic features being extracted, for a total 1,820 radiomic features extracted [21]. The preprocessing steps are outlined in the parameter file available in the GitHub repository [27].

### Feature selection

Optimal radiomic features were selected using an unsupervised machine learning approach (Fig. 2) to identify a subset with strong predictive potential prior to model building. A correlation filter was applied to all the 1,820 extracted radiomic features, eliminating features with a Pearson correlation > 0.80 in absolute value. A minimum variance filter, Near Zero Variance, was applied to the remaining features to exclude any noninformative variables. As radiomic features are susceptible to variation between different MRI scanners [28], the distributions of the remaining features from each of the two MRI scanners used in this study were compared by way of a two-sample, two-sided Mann–Whitney *U* test [29] at the 5% significance level; *p* values from these multiple tests were corrected for false discovery rate (FDR) [30]. Features with high cross-scanner discrepancies, defined as an adjusted *p* value < 0.05, were removed. A test–retest analysis was performed on the remainder set of features to evaluate stability of the radiomic feature quantitation with respect to inter-reader variability for a subset of 22 cases; the minimum Pearson correlation between features extracted from the segmentations performed by different readers on the same cases was 0.75, and the 5th percentile of Pearson correlations was 0.969. For intraclass correlation (ICC), similar findings were found with a 5th ICC percentile of 0.965.

### Predictive modelling

The following clinical variables were included for modelling: body mass index, maternal age, and the number of prior Caesarean sections. Differences between clinical variables and radiomic features were compared between FIGO grade 1–2 and grade 3 cases using a two-sample, two-sided Mann–Whitney *U* test. After *p* value adjustment for FDR, there were no significant associations, with a smallest *p* value of 0.228.

Univariate logistic regression models trained using bootstrapping, and tested on the out-of-bag bootstrap points, were used to identify the best performing radiomic features. The performance of univariate models was assessed on the basis of the area under curve (AUC) at receiver operating characteristic (ROC) analysis and of overall prediction accuracy. Four multivariate classifiers were trained for PAS prediction: least absolute shrinkage and selection operator (LASSO), random forest (RF), k-nearest neighbour, and support vector machine (SVM) [31]. Their performance was assessed using calibration curves, ROC analysis, sensitivity, specificity and accuracy. As three of these models (namely LASSO, RF, and SVM) include a built-in feature selection mechanism, final predictive feature sets were also analysed from these modelling pipelines.

One-sided, two-sample Mann–Whitney *U* tests were carried out to compare performance metrics between univariate and multivariate models at the 5% significance level after FDR correction. These tests were unpaired since the models were bootstrapped separately, each using different resamples.

Variable importance can be measured for these multivariate models to determine the extent of the contribution from each feature, and were thus analysed. In complement to these metrics, Principal component analysis (PCA), a dimensionality reduction technique used to explain the total variation (*i.e.*, information) in the dataset along principal components arranged in decreasing order of relevance [32], was further performed to explore how the final feature sets summarised the overall information available for analysis. Biplots of the PCA-based projections of the final predictive feature sets were considered to assess the level of redundancy present in the latter, and possibly identify key features driving the prediction.

### Statistical software

Statistical analysis for this study was performed in RStudio (version 4.2.2 [33]). Feature reduction and model building were performed using R with the caret [34], pROC [35], and corrplot [36] packages.

## Results

Forty-one participants met inclusion criteria (34 from site 1, 7 from site 2), including 18 FIGO grade 1–2 and 23 FIGO grade 3 PAS cases. Participants had a median age of 37.0 years (interquartile range 34.0 × 40.0 years) and were predominantly of white Irish ethnicity (Table 1). All women had at least one prior Caesarean section, and 29/41 (70.7%) underwent caesarean hysterectomy.

**Table 1:** Participant demographics and clinical outcome

	FIGO grade 1–2 (*n* = 18)	FIGO grade 3 (*n* = 23)
Age (years)	36.0 (34.0–39.75)	39.0 (37.2–42.7)
Body mass index (kg/m^2^)	25.2 (23.4–29.3)	25.7 (23.0–30.1)
Parity	2 (1–3)	2 (1–2.5)
No of previous Caesarean section	1 (1–2)	2 (1–3)
Gestation at MRI (weeks + days)	29 + 0 (27 + 2 − 32 + 3)	28 + 1 (27 + 0–31 + 0)
Placental location on MRI, *n* (%)		
Placenta previa	18 (100.0)	22 (95.6)
*Anterior placenta previa*	16 (88.0)	22 (95.6)
*Posterior placenta previa*	2 (12.0)	0 (0.0)
Elective delivery, *n* (%)	30 (75.0)	7 (100.0)
Estimated blood loss (mL)	1,100 (735–3,250)	1,600 (1,100–5,800)
Red cell concentrate transfusion, *n* (%)	6 (33.3)	10 (43.5)
Surgical outcome		
Caesarean hysterectomy, *n* (%)	7 (38.9)	22 (95.7)
Uterine conservation^a^ (%)	11 (61.6)	1 (4.3)
FIGO histological grade, *n* (%)		
1	4 (22.2)	0 (0.0)
2	14 (77.8)	0 (0.0)
3	0 (0.0)	23 (100)

Data are given in median (interquartile interval) unless otherwise stated

^a^Uterine conservation for the PAS group were cases who underwent myometrial resection. *FIGO* International Federation of Gynecology and Obstetrics

The feature filtering steps of correlation filter and Near Zero Variance yielded a subset of 47 features from the original images, and a subset of 225 features when including any features from either original and pre-filtered images. Following removal of features with high cross scanner discrepancies, a final feature set of 204 features were selected.

### Univariate analyses

**Fig. 3:**
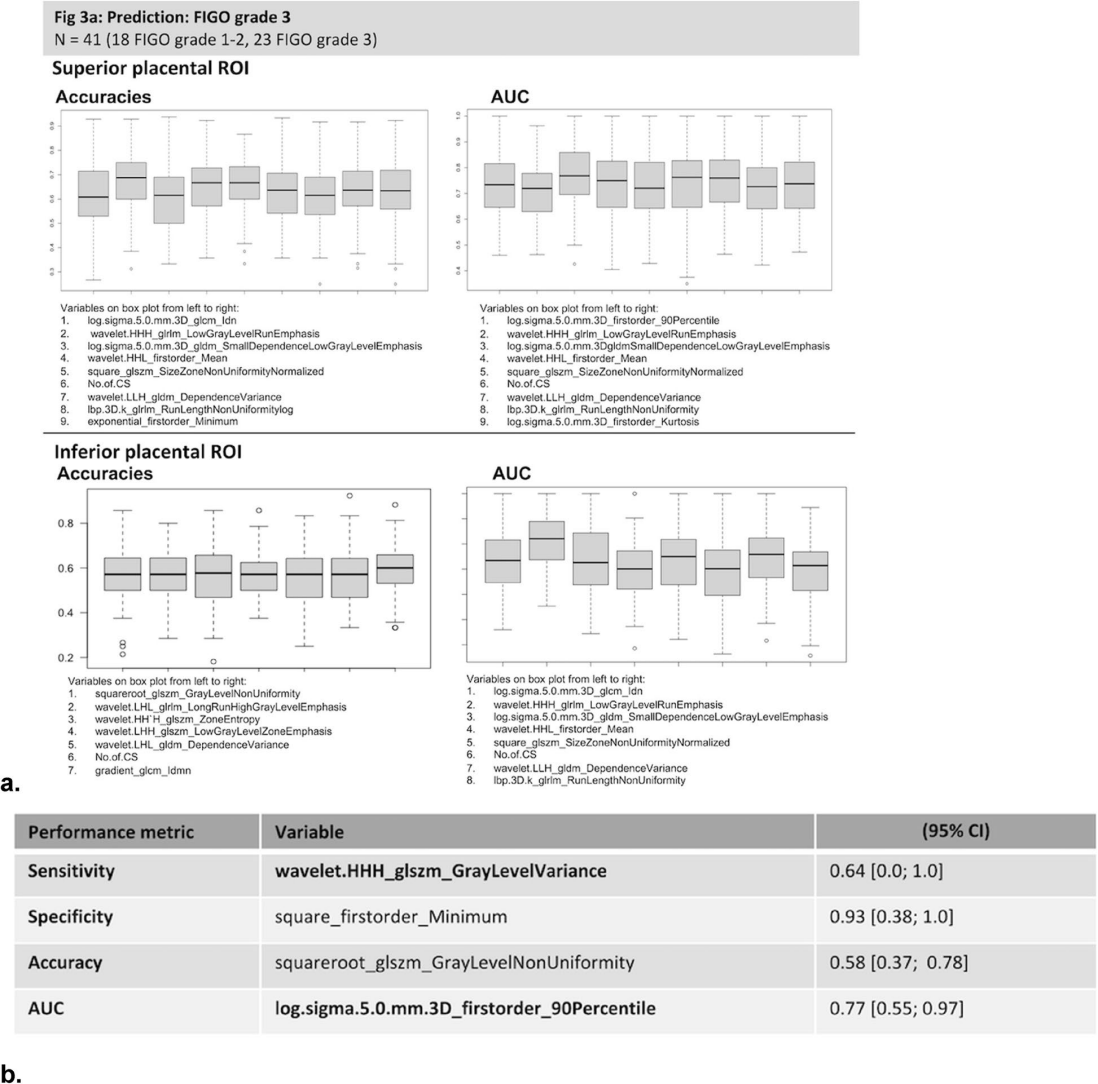
Univariate bootstrapped linear regression models. a Box-plots showing performance metrics of models as estimated by accuracy, area under the curve (AUC) for top performing radiomic features. b Radiomic features with top performance metrics from univariate analysis; the table lists the variables with the highest performance for each performance metric of sensitivity, specificity, accuracy, and AUC from the univariate bootstrapped linear regression analysis (in bold, superior placental region of interest)

The radiomic features identified by univariate analysis as most strongly associated with FIGO grade 3 PAS are shown in Fig. 3. Clinical features included in the models were not of high importance, with only the number of previous Caesarean section included in the top ten accuracies or AUC from univariate logistic regression (Fig. 3).

From the superior placental ROI, exponential_glszm_SizeZoneNonUniformityNormalized yielded the highest bootstrapped accuracy of 0.58 (95% confidence interval [CI] 0.39−0.77) and the highest AUC was 0.77 (0.56−0.97]) from log.sigma.5.0.mm.3D_firstorder_90Percentile. The highest sensitivity was wavelet.HHH_glszm_GrayLevelVarianceof 0.64 (0.0−1.00]), while specificity was 0.88 (0.40−1.00]) from lbp.3D.m2_firstorder_Range. For the inferior placental ROI, squareroot_glszm_GrayLevelNonUniformity yielded the highest accuracy of 0.58 (0.37−0.78) and a highest AUC 0.75 (0.56−0.94) from log.sigma.2.0.mm.3D_firstorder_Maximum. A sensitivity of 0.62 (95% CI: [0.61; 1]) was achieved from wavelet.HHH_firstorder_Median, and a highest specificity of 0.93 (0.38−1.00]) from square_firstorder_Minimum (Fig. 3b).

At univariate analysis, the inferior and superior placental ROIs yielded similar levels of performance (Fig. 3a). Subsets of individual radiomic features with high specificity were found in both regions, while sensitivity was poorer for both. The radiomic profiles of inferior and placental regions were, however, different with little correlation between them (Pearson ρ−0.26 and + 0.31). Thirty per cent of features were significantly different between the regions (19.4% were significantly greater in the superior ROI, and 11.6% in the inferior ROI) at the 5% significance level based on one-sided, two-sample Mann–Whitney *U* tests. These results indicate that high specificity was facilitated by different radiomic profiles in the inferior and superior placental areas.

### Multivariate predictive modelling

**Fig. 4:**
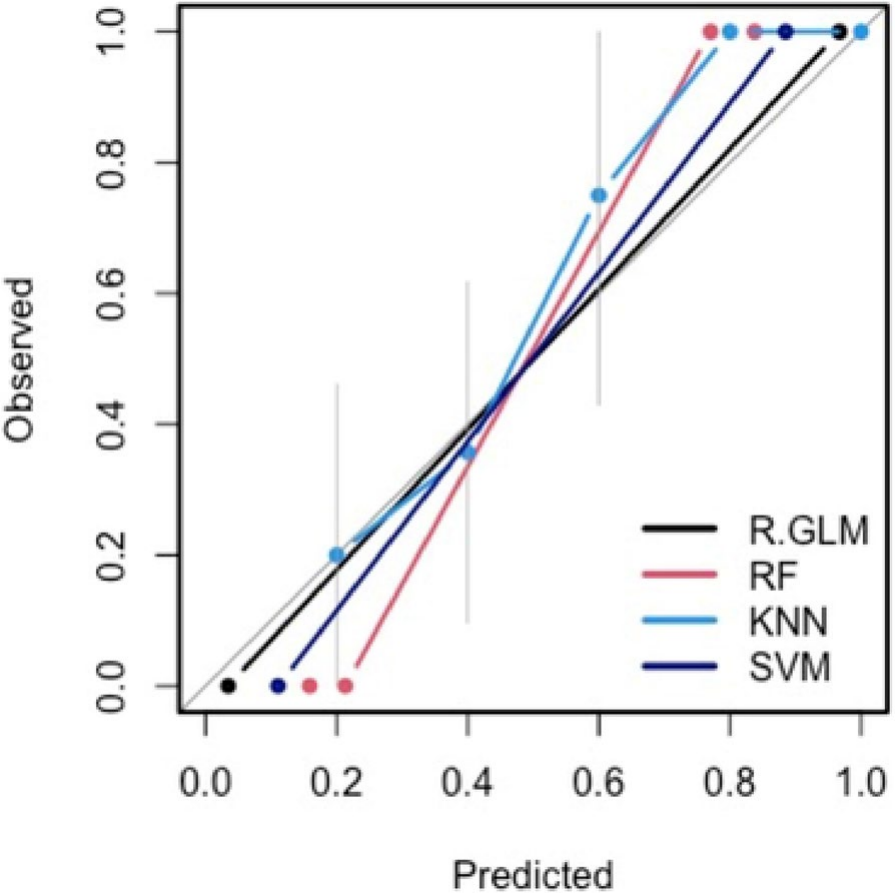
Model calibration curves with associated 95% confidence bands (grey). The *y*-axis represents the actual probability, and the *x*-axis represents the predicted probability of placenta accreta spectrum. Each curve corresponds to a predictive model and assesses the alignment between mean estimated model probabilities obtained from that model, and observed event rates within each risk group. Here the risk groups are defined with respect to the quintiles of the predicted probabilities from that model. The closer the lines are to the ideal grey line (45° line), the better the prediction accuracy of the model

**Fig. 5:**
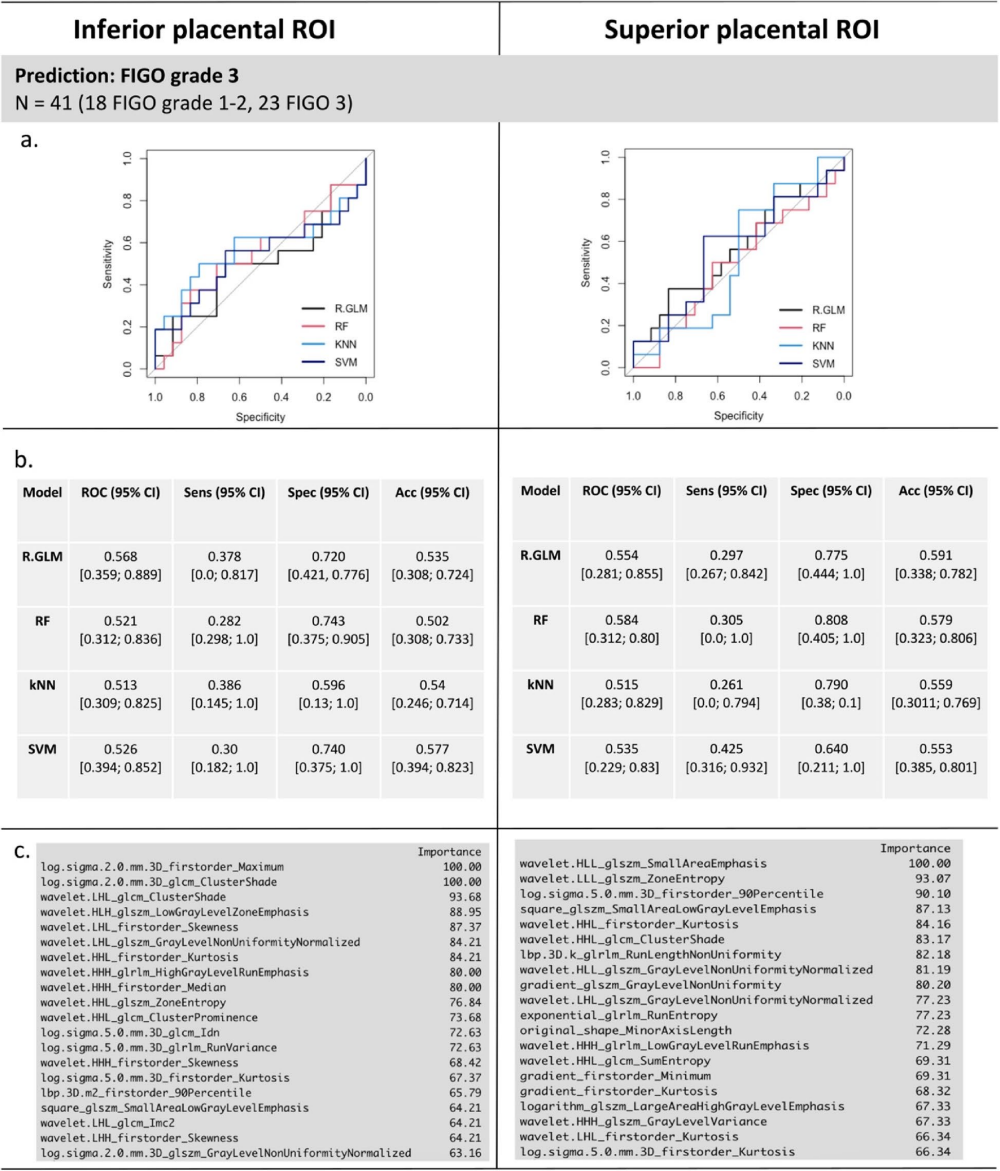
Multivariate bootstrapped models. The performance of each model for predicting severe FIGO grade 3 PAS from the inferior and superior placental ROI is shown. In panels **a** and **b**, the ROC curve and performance metrics for each of the models for predicting invasive FIGO grade 3 PAS are shown. Panel **c** reports the variable importance for radiomic and clinical features used in the prediction for SVM from the inferior and superior placental ROIs. *CS* Caesarean section, *FIGO* International Federation of Gynecology and Obstetrics, kNN: k-nearest neighbour, *PAS* Placenta accreta spectrum, *R-GLM LASSO*, Least absolute shrinkage and selection operator, *RF* Random forest, *ROI* Region of interest, *SVM* Support vector machine

For classification based on the four multivariate models (LASSO, RF, kNN, and SVM), calibration curves demonstrated reasonable agreement between the predicted and observed rates of FIGO grade 3 PAS, indicating the models estimate the probability of PAS appropriately both for FIGO grade 3 and non-grade 3 cases (Fig. 4). Models had a similar performance overall for predicting FIGO grade 3 PAS, with all models having a ROC of above 50% and specificity above 60% (Fig. 5a, b). Comparing the inferior and superior placental ROIs, RF had a significantly higher accuracy in the superior compared to the inferior placental ROI (Mann–Whitney *U* test, *p* = 0.003), while kNN had a significantly greater specificity in the inferior region *versus* superior region (Mann–Whitney *U* test, *p* = 0.001). There were no other significant differences between model performance between the inferior and superior ROIs (all *p* values > 0.05, Supplementary Figure S1).

The variables of highest importance for multivariate modelling are shown in Fig. 5c; radiomic features were ranked as highest importance, with no clinical features ranked as important for modelling. For multivariate models, radiomic features with image filters applied were selected at higher frequency than those from the original image, with no radiomic feature from the original image feature ranked in the top 20 predictive features for either the inferior and superior placental ROIs for predicting severe FIGO grade 3 PAS. Features from various families were included in the final feature sets for prediction, including first-order, Gray Level Run Length Matrix, Gray Level Size Zone Matrix, and Gray Level Dependence Matrix.

**Fig. 6:**
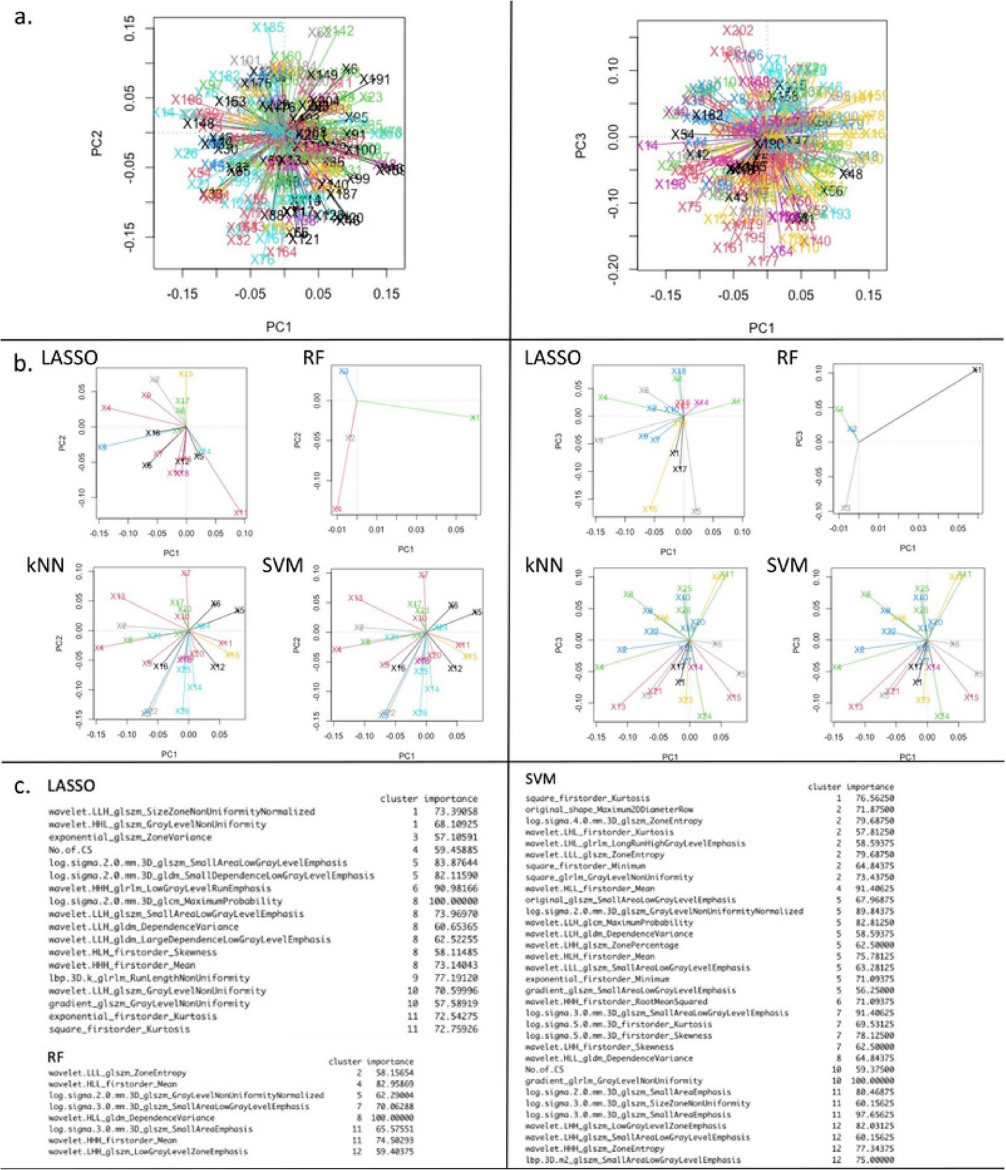
Principal component analysis (PCA) from superior placental region of interest using all radiomic features from image filters. Panel **a** shows how the feature space used by each model from whole dataset. The plots show each model is using information for making predictions from different areas of the feature space. Panel **b** shows PCA for each model. Panel **c** shows the radiomic features within each PCA cluster that were important for each prediction model. This suggests some radiomic features could be used either interchangeably or in combination for placenta accreta spectrum prediction. **kNN yielded variable importance and PCA outputs identical to those obtained from support vector machine (SVM) as seen in panel **b** (due to the discretisation method used in evaluating variable importance for these two models) and therefore only the output for SVM are shown in **c**

PCA was then performed to further explore the structure of final feature sets obtained from each model (Fig. 6), in particular to analyse how the final predictors tend to group up into separate predictive clusters. To do this, the distribution in the PCA domain of all features used for model training was first analysed using k-means clustering [31]. Then, the final feature sets obtained from each model were mapped to these clusters in order to identify high-predictive clusters of features, and relevant features within these specific clusters. From this analysis, it was observed that all models contained the first-order mean level from the wavelet HLL-filtered image in their final feature subset. k-nearest neighbour and SVM selected the same feature subsets, due to the discretisation method used in evaluating variable importance for these two models, which included only one clinical variable, *i.e.*, the number of prior Caesarean sections. Both these models tended to use more information from some of the clusters in particular, *i.e.*, groups of predictors with a high level of information overlap.

## Discussion

We presented a methodological approach and results from a bi-centre study for radiomics-based prediction of disease severity for PAS. This work contributes a radiomics study employing standardised clinical-histopathological descriptions for PAS [2, 15]. Furthermore, radiomic feature extraction was performed using well described methods, with the steps of feature reduction and predictive modelling provided as open source code. We found a high specificity of radiomics-based prediction for severe FIGO grade 3 PAS cases.

Our outcome for predictive modelling was histopathological diagnosis, the gold standard for diagnosis of PAS [2, 15]. Previous studies have used radiomic features to predict clinical outcomes such as undergoing caesarean hysterectomy and blood loss [13, 37], however, we opted to build our models using only the histopathological FIGO grading of PAS severity. This was because clinical outcomes, such as performing a caesarean hysterectomy, reflect the local clinical practice rather than the suspected severity of disease from antenatal imaging. For example, centres practicing conservative management with leaving the placenta *in situ* may have much lower hysterectomy rates compared to centres where hysterectomy is routinely performed for PAS cases [22, 38]. Similarly, using haemorrhage as an outcome for predicting modelling is limited as blood loss measurement is highly heterogenous and often inaccurate, hence complicating comparisons between studies using this as an outcome [39, 40]. Moreover, clinical outcomes are often not related to disease severity, but to other factors such as emergency delivery, as well as clinician consideration of the woman’s parity, her preferences and future fertility plans [3, 41]. Hence, we choose to develop our models on a reproducible histopathological outcome reflective of disease severity.

We found radiomic features from both placental ROIs had an overall similar performance. We hypothesised that radiomic features from the inferior placental ROI would be more predictive of FIGO grade 3 PAS as this is the area where abnormal uterine remodelling from a prior Caesarean section has resulted in abnormal placentation [42]. Our results suggests there are placental changes beyond the myometrial-bladder interface, which were useful for classifying severe PAS cases. Well-described MR imaging features, such as abnormal vascularisation of the placental bed and focal exophytic mass, are signs of severe PAS; they are usually seen at the level of the bladder, and are therefore included in the inferior placental ROI [5]. MRI features such as placental heterogeneity and T2-dark bands are seen throughout the whole placenta. Other features of severe PAS, such as intraplacental fetal vessel diameter, are also evident throughout the placenta [43]. These appear as darkened areas deep in the placenta. Hence, it follows that the most predictive features from the superior placental ROI were textural features describing differences in homogeneity and grey level values. This suggests that in PAS there may be textural changes throughout the whole placenta that were useful for our modelling.

We report the use of image pre-processing and use of pre-processing image filters resulted in significantly better model performance compared to use of the original image features only. There is currently no consensus on how to perform these steps for MR images in radiomics, and is one of the many open challenges in radiomic processing [18, 44]. Our results support the finding of previous radiomic studies in PAS, where radiomic features from image filters were more predictive and had higher diagnostic accuracy than those from the original image [45, 46]. Previously, some studies reported the use of pre-processing image filters was shown to improve predictive performance of models [47, 48]. However, when datasets are small, limiting the analysis to radiomic features from the original image only showed similar results to when all radiomic features with image filters were included [47]. Here we also performed predictions using both the original image features and using all features from image filters. However, we found that features from the pre-processed images had better predictive potential.

Initiatives such as the RQS [20] and Image Biomarker Standardisation Initiative [49] attempt to harmonise radiomic studies and produce results which can be validated and applied to clinical settings. Few radiomic studies to date have included a RQS in their work [20], and many when assessed externally score poorly, with the studies in the systematic review on PAS radiomics having a median RQS score of 23% [14].

The RQS of this study was 38% (see Supplementary materials), however, this score was developed to assess the quality of oncologic radiomics studies and not studies exploring conditions such as PAS. This study, as with many studies not assessing oncologic data, is penalised by the RQS in a number of areas. Firstly, image data is not available at multiple time points. In oncology, the purpose of repeat imaging is to assess response to a treatment, such as chemotherapy. In PAS, the condition is limited to a distinct period of time—pregnancy—and once the pregnancy is completed and the placenta removed, the condition can be considered as “treated”. Hence, unlike in cancer care, there is no indication to repeat imaging to assess treatment response.

Secondly, points are assigned for using open source images, segmentations and code. To our knowledge, these are not currently available for PAS. Any future such dataset would need to ensure the inclusion criteria are as described by the FIGO classification. For segmentations, while automated segmentations may be considered the ideal approach as it minimises the inter-observability between segmentations, the applicability of trained algorithms to new datasets is currently limited and often results in failure of accurate segmentations [18]. Attempts to limit inter-observer variability in this study included calculation of the ICC between readers for a subset of segmentations, a clearly defined segmentation protocol and three investigators independently performing segmentations with cross-reference. If a disagreement arose, the third investigator acted as mediator.

PAS remains a rare complication of pregnancy, however the incidence is increasing as a result of the rise in the Caesarean section rate [50]. Ultrasound and MRI both rely heavily on reader expertise of the clinician [5, 10]. In this study, radiomic features had a reasonable sensitivity for identifying severe FIGO grade 3 cases on univariate analysis, and a high specificity. This demonstrates the clinical potential of using radiomics to detect and rule out severe PAS. By identifying severe PAS subtypes, interventions associated with additional morbidity, such as elective preterm delivery and interventional radiology techniques, may be reserved for these cases. While these findings will require validation in an external dataset, this work supports the potential use of radiomics in predicting disease severity in PAS.

This study has several limitations. Firstly, the sample size is small. Nonetheless, results from our pilot dataset demonstrated that radiomic features predicted severe PAS. Efforts to increase our sample size were employed, by using data available from another centre. As a result of the small sample size, it is not surprising that multivariate models showed a drop in performance compared to univariate analysis given the loss of statistical power with a small N:P ratio. Some important MRI features of PAS such as myometrial thinning and interruption of the bladder wall will not be captured by the ROIs presented here, which include only the placenta. However, there was consensus that there would be limited accuracy in delineating the myometrium which in many cases will be thinned to less than 1 mm or not be visible [5]; hence, only the placenta was segmented for this work. Although segmentations were performed manually by three readers, radiomic features were very stable across the readers as demonstrated by high Pearson correlation on test–retest analyses.

In summary, we present a suggested methodology for MRI segmentation and radiomic processing for predicting disease severity in PAS. Despite the restrictive size of the dataset, we found radiomic features have the potential predict severe FIGO grade 3 PAS cases. Radiomics to predict disease severity may assist clinicians in individualising care for women with PAS. Future studies can implement the prediction model using larger datasets to validate and improve upon the results reported here.

### Supplementary Information

**Additional file 1.**

## Data Availability

The code used for the methods presented in this manuscript are provided as open source in a public repository. The image data used for analysis is not publicly available.

## Abbreviations

AUC: Area under the curve
CI: Confidence interval
FDR: False discovery rate
FIGO: International Federation of Obstetrics and Gynaecology
ICC: Intraclass correlation
LASSO: Least absolute shrinkage and selection operator
MRI: Magnetic resonance imaging
PAS: Placenta accreta spectrum
PCA: Principal component analysis
RF: Random forest
ROC: Receiver operating characteristic
ROI: Region of interest
RQS: Radiomics Quality Score
SVM: Support vector machine
