# Correction: Radiomics-based prediction of FIGO grade for placenta accreta spectrum

**DOI:** 10.1186/s41747-023-00397-y

**Published:** 2023-11-22

**Authors:** Helena C. Bartels, Jim O’Doherty, Eric Wolsztynski, David P. Brophy, Roisin MacDermott, David Atallah, Souha Saliba, Constance Young, Paul Downey, Jennifer Donnelly, Tony Geoghegan, Donal J. Brennan, Kathleen M. Curran

**Affiliations:** 1 Department of UCD Obstetrics and Gynaecology, School of Medicine, University College Dublin, National Maternity Hospital, Holles Street, Dublin 2, Ireland; 2 Siemens Medical Solutions, Malvern, PA USA; 3 Department of Radiology & Radiological Science, Medical University of South Carolina, Charleston, SC USA; 4 Radiography & Diagnostic Imaging, University College Dublin, Dublin, Ireland; 5 Statistics Department, University College Cork, Cork, Ireland; 6 Insight Centre for Data Analytics, Dublin, Ireland; 7 Department of Radiology, St. Vincent’s University Hospital, Dublin, Ireland; 8 Department of Gynecology and Obstetrics, Hôtel-Dieu de France University Hospital, Saint Joseph University, Beirut, Lebanon; 9 Department of Radiology: Fetal and Placental Imaging, Hôtel-Dieu de France University Hospital, Saint Joseph University, Beirut, Lebanon; 10 Department of Histopathology, National Maternity Hospital, Dublin, Ireland; 11 Department of Obstetrics and Gynaecology, Rotunda Hospital, Dublin, Ireland; 12 Department of Radiology, Mater Misericordiae University Hospital, Dublin, Ireland; 13 University College Dublin Gynaecological Oncology Group (UCD-GOG), Mater Misericordiae University Hospital and St Vincent’s University Hospital, Dublin, Ireland; 14 Systems Biology Ireland, School of Medicine, University College Dublin, Dublin, Ireland; 15 School of Medicine, University College Dublin, Dublin, Ireland

**Correction: European Radiology Experimental 7, 54 (2023)**

**https://doi.org/10.1186/s41747-023-00369-2**

The original article [1] contains numerical errors in Table 1.

**Table 1:** Participant demographics and clinical outcome

	FIGO grade 1–2 (*n* = 18)	FIGO grade 3 (*n* = 23)
Age (years)	36.0 (34.0–39.75)	39.0 (37.2–42.7)
Body mass index (kg/m^2^)	25.2 (23.4–29.3)	25.7 (23.0–30.1)
Parity	2 (1–3)	2 (1–2.5)
No of previous Caesarean section	1 (1–2)	2 (1–3)
Gestation at MRI (weeks + days)	29 + 0 (27 + 2 − 32 + 3)	28 + 1 (27 + 0–31 + 0)
Placental location on MRI, *n* (%)		
Placenta previa	18 (100.0)	22 (95.6)
*Anterior placenta previa*	16 (88.0)	22 (95.6)
*Posterior placenta previa*	2 (12.0)	0 (0.0)
Elective delivery, *n* (%)	13 (72.0)	17 (73.0)
Estimated blood loss (mL)	1,100 (735–3,250)	1,600 (1,100–5,800)
Red cell concentrate transfusion, *n* (%)	6 (33.3)	10 (43.5)
Surgical outcome		
Caesarean hysterectomy, *n* (%)	7 (38.9)	22 (95.7)
Uterine conservation^a^ (%)	11 (61.6)	1 (4.3)
FIGO histological grade, *n* (%)		
1	4 (22.2)	0 (0.0)
2	14 (77.8)	0 (0.0)
3	0 (0.0)	23 (100)

Data are given in median (interquartile interval) unless otherwise stated

^a^Uterine conservation for the PAS group were cases who underwent myometrial resection

*FIGO *International Federation of Gynecology and Obstetrics

In the row "Elective delivery, n (%)", the respective cells state '30 (75)' and '7 (100)'.

They should instead respectively state '13 (72.0)' and '17 (73.0)' as shown in Table 1 of this Correction article.
