# Supplementary material for: Radiomics-based prediction of FIGO grade for placenta accreta spectrum
Source: Eur Radiol Exp. 2023 Sep 20;7:54. doi: 10.1186/s41747-023-00369-2 (PMC10509122; doi:10.1186/s41747-023-00369-2)
Supplement: Supplementary file 1 — Additional file 1. [file 41747_2023_369_MOESM1_ESM.pdf]

Radiomics-based prediction of FIGO grade for placenta accreta spectrum

ELECTRONIC SUPPLEMENTARY MATERIAL

1. **Annotation Protocol for PAS Radiomics work**
2. **Figure S1: box-plots comparing model performance from multivariate analysis for inferior and superior ROIs**
3. **TRIPOD checklist**
4. **Radiomics Quality Score**

1

**Annotation Protocol for PAS Radiomics work**			
**Slice**	Balanced steady state free procession<br>(b-SSFP) sequence, sagittal plane<br>Identify ~mid sagittal slice where internal os is seen (mark internal os)<br>Measure 6cm from internal os (<6cm internal os = inferior, >6cm from internal os = superior ROI)<br>Orthogonal line to separate placenta into superior and inferior ROI Generate 2 placental ROIs (superior, inferior) on each slice<br>Mid slice with internal os = slice number 5<br>Slices 1,2,3,4 = to right side of mid slice<br>5 “liver side”<br>Slices 6,7,8,9 = to left side of mid slice 5<br>“spleen side”	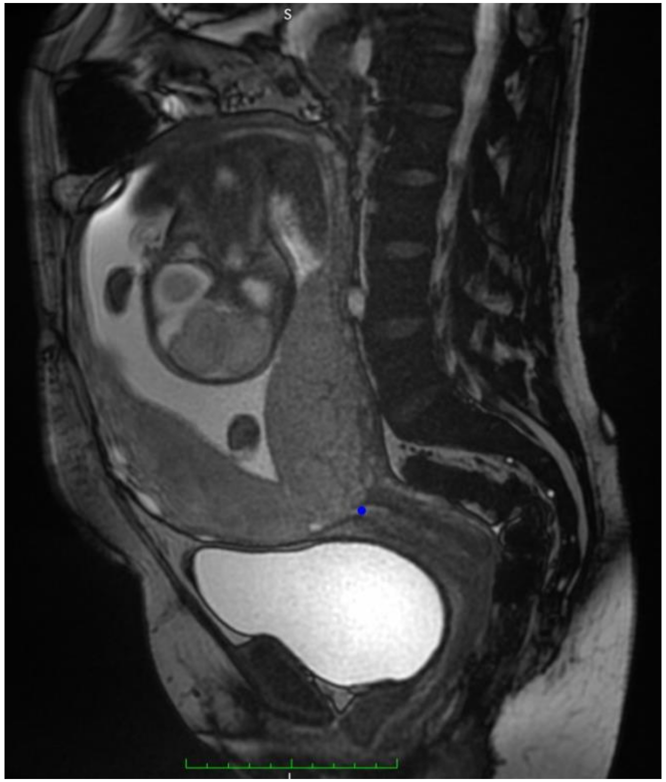	Sagittal, b-SSFP sequence, of PAS case at 30 weeks’ gestation. The internal cervical os is marked using point annotation tool in Osirix.

**Annotations**	ROIs are generated in Osirix using the following tools: “point”, “closed polygon” and “length”<br>Internal os: point, mid thickness, blue<br>Orthogonal line dividing placenta into superior/inferior: 6cm from internal os.<br>Orthogonal line	
	Superior placenta ROI: closed polygon, magenta<br>Inferior placenta ROI: closed polygon, yellow	

**Summary of steps**	1. Open MR sequence in Osirix<br>2. Choose b-SSFP sagittal view<br>3. Identify internal cervical os in the mid sagittal plane<br>4. Label internal cervical os with blue point (label internal os)<br>5. Measure 6cm from internal cervical os<br>6. Divide placenta into two regions superior and inferior to internal os at<br>6cm marker with orthogonal line 7. Draw ROI superior and inferior placenta with closed polygon tool. Label two areas<br>8. Label 4 slices to right and left of central slice<br>9. Slices to the right = slice numbers<br>1,2,3,4<br>10. Slices to the left = slice numbers<br>6,7,8,9<br>11. Save all 9 annotated slice images as<br>above: study ID_slice number	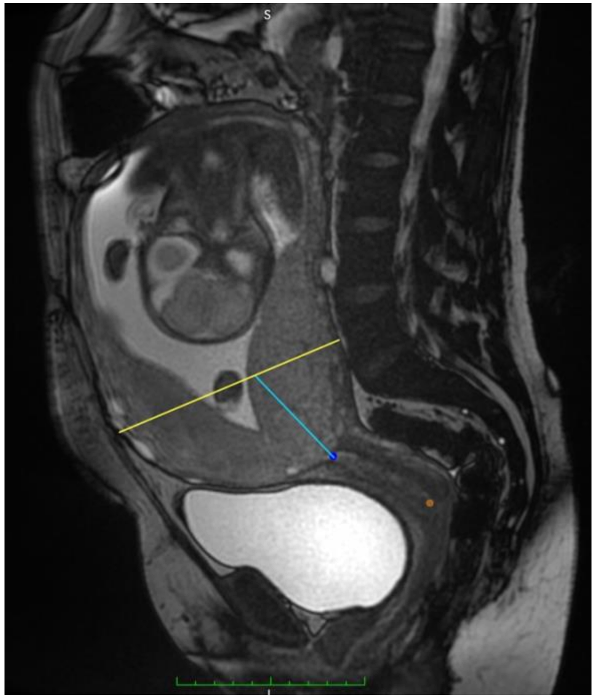 Steps 1-6: internal cervical os marked, orthogonal line 6cm from internal cervical os (blue line), orthogonal line to divide placenta (yellow line) into area proximal to area of placental adherence or invasion (inferior placenta) and distal from this area (superior placenta).

		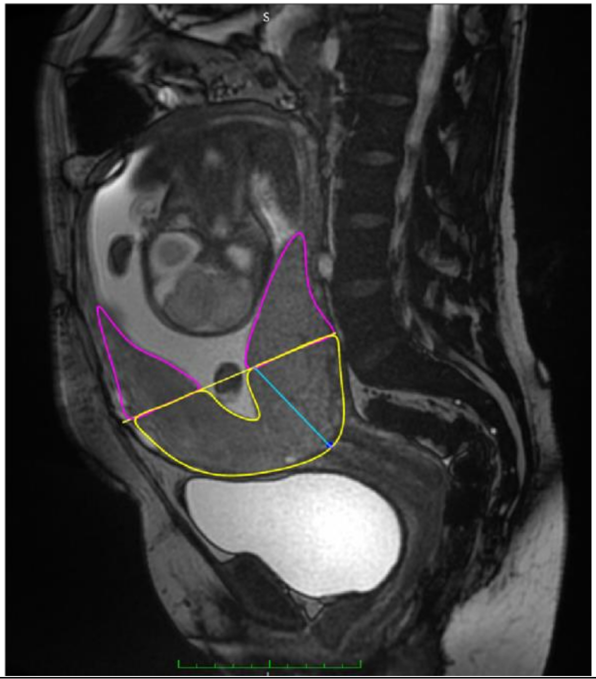	Step 7 : superior and inferior placenta ROIs generated using closed polygon tool.

**File preparation for radiomic feature extraction**	1. Osirix ROIs are exported as JSON files using “export ROI plugin”.<br>2. In 3D slicer, MRI for the corresponding case is opened.<br>3. The following 3D slicer extensions were installed to facilitate ROI importation from Osirix and radiomic feature extraction: SlicerRadiomics, SandBox and Slicer RT.<br>4. Segmentations are imported into 3D slicer using “Import Osirix ROI” tool.<br>5. The MRI and the segmentations are exported as NRRD. files using “Segmentations” function in 3D slicer.<br>6. A CSV file is created with paths to the image file and corresponding mask for each case using the NRRD. file paths.	OSIRIX ROIS 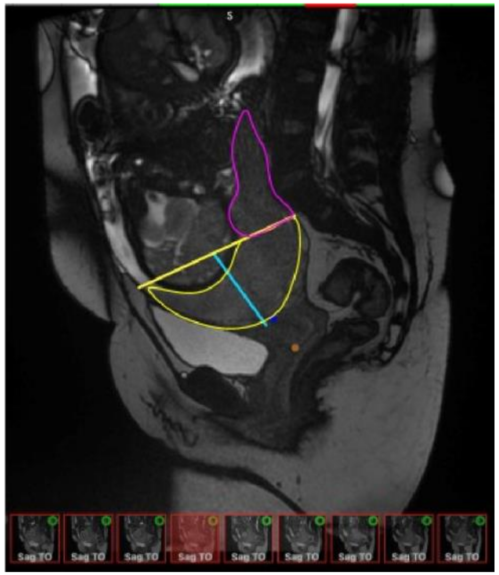 à 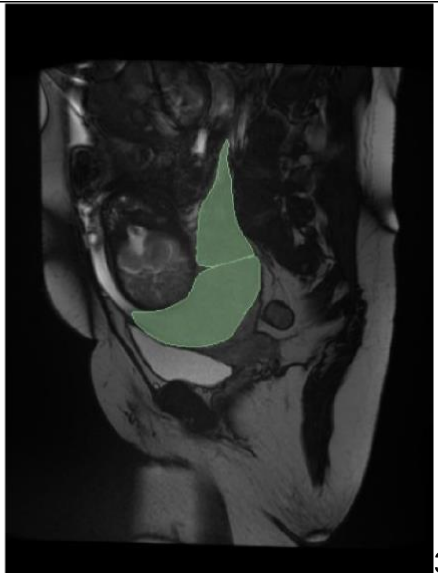 3D SLICER<br>Segmentations created in Osirix (left) are exported as JSON files, and imported into 3D slicer (right) to create NRRD. files for radiomic feature extraction.

Fig S1: box-plots comparing model performance from multivariate analysis for inferior and superior ROI

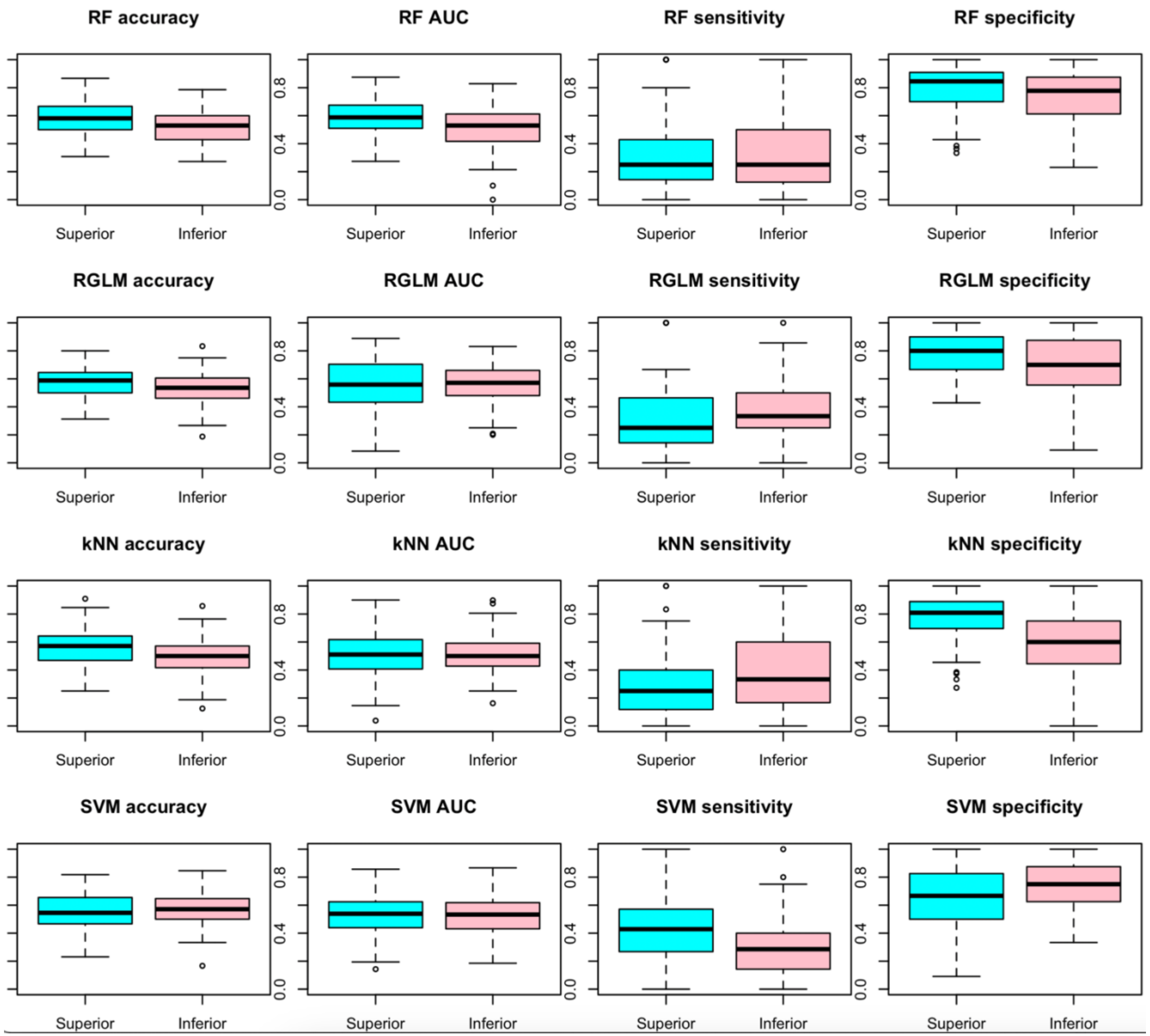

3

TRIPOD Checklist: Prediction Model Development

Section/Topic	m	Checklist Item	Page
**Title and abstract**			
Title	1	Identify the study as developing and/or validating a multivariable prediction model, the target population, and the outcome to be predicted.	1
Abstract	2	Provide a summary of objectives, study design, setting, participants, sample size, predictors, outcome, statistical analysis, results, and conclusions.	4
**Introduction**			
Background and objectives	3a	Explain the medical context (including whether diagnostic or prognostic) and rationale for developing or validating the multivariable prediction model, including references to existing models.	6-8
	3b	Specify the objectives, including whether the study describes the development or validation of the model or both.	8
**Methods**			
Source of data	4a	Describe the study design or source of data (e.g., randomized trial, cohort, or registry data), separately for the development and validation data sets, if applicable.	9
	4b	Specify the key study dates, including start of accrual; end of accrual; and, if applicable, end of follow-up.	9

Participants	5a	Specify key elements of the study setting (e.g., primary care, secondary care, general population) including number and location of centres.	9
	5b	Describe eligibility criteria for participants.	9
	5c	Give details of treatments received, if relevant.	23
Outcome	6a	Clearly define the outcome that is predicted by the prediction model, including how and when assessed.	9
	6b	Report any actions to blind assessment of the outcome to be predicted.	9-11
Predictors	7a	Clearly define all predictors used in developing or validating the multivariable prediction model, including how and when they were measured.	9-11
	7b	Report any actions to blind assessment of predictors for the outcome and other predictors.	9-11
Sample size	8	Explain how the study size was arrived at.	14
Missing data	9	Describe how missing data were handled (e.g., complete-case analysis, single imputation, multiple imputation) with details of any imputation method.	12-13
Statistical analysis methods	10a	Describe how predictors were handled in the analyses.	12-13
	10b	Specify type of model, all model-building procedures (including any predictor selection), and method for internal validation.	12-13
	10d	Specify all measures used to assess model performance and, if relevant, to compare multiple models.	12-13
Risk groups	11	Provide details on how risk groups were created, if done.	
**Results**			
Participants	13a	Describe the flow of participants through the study, including the number of participants with and without the outcome and, if applicable, a summary of the follow-up time. A diagram may be helpful.	14
	13b	Describe the characteristics of the participants (basic demographics, clinical features, available predictors), including the number of participants with missing data for predictors and outcome.	14
Model development	14a	Specify the number of participants and outcome events in each analysis.	14, 23

	14b	If done, report the unadjusted association between each candidate predictor and outcome.	
Model specification	15a	Present the full prediction model to allow predictions for individuals (i.e., all regression coefficients, and model intercept or baseline survival at a given time point).	Figures
	15b	Explain how to the use the prediction model.	
Model performance	16	Report performance measures (with CIs) for the prediction model.	14-15
**Discussion**			
Limitations	18	Discuss any limitations of the study (such as nonrepresentative sample, few events per predictor, missing data).	19-20
Interpretation	19b	Give an overall interpretation of the results, considering objectives, limitations, and results from similar studies, and other relevant evidence.	20
Implications	20	Discuss the potential clinical use of the model and implications for future research.	20
**Other information**			
Supplementary information	21	Provide information about the availability of supplementary resources, such as study protocol, Web calculator, and data sets.	Github link page 10
Funding	22	Give the source of funding and the role of the funders for the present study.	3

Image protocol quality - well-documented image protocols (for example, contrast, slice thickness, energy, etc.) and/or usage of public image protocols allow reproducibility/replicability protocols well documented public protocol used none<br>☑<br>☐<br>☐
Multiple segmentations - possible actions are: segmentation by different physicians/algorithms/software, perturbing segmentations by (random) noise, segmentation at different breathing cycles. Analyse feature robustness to segmentation variabilities yes no<br>◉<br>○
Phantom study on all scanners - detect inter-scanner differences and vendor-dependent features. Analyse feature robustness to these sources of variability yes no<br>○<br>◉
Imaging at multiple time points - collect images of individuals at additional time points. Analyse feature robustness to temporal variabilities (for example, organ movement, organ expansion/shrinkage) yes no<br>○<br>◉
Feature reduction or adjustment for multiple testing - decreases the risk of overfitting. Overfitting is inevitable if the number of features exceeds the number of samples. Consider feature robustness when selecting features<br>◉ Either measure is implemented<br>○ Neither measure is implemented
Multivariable analysis with non radiomics features (for example, EGFR mutation) - is expected to provide a more holistic model. Permits correlating/inferencing between radiomics and non radiomics features yes no<br>◉<br>○
Detect and discuss biological correlates - demonstration of phenotypic differences (possibly associated with underlying gene–protein expression patterns) deepens understanding of radiomics and biology yes no<br>○<br>◉

Cut-off analyses - determine risk groups by either the median, a previously published cut-off or report a continuous risk variable. Reduces the risk of reporting overly optimistic results ○ yes

◉ no

Discrimination statistics - report discrimination statistics (for example, C-statistic, ROC curve, AUC) and their statistical significance (for example, p-values, confidence intervals). One can also apply resampling method (for example, bootstrapping, cross-validation)

- [x] a discrimination statistic and its statistical significance are reported
- [x] a resampling method technique is also applied
- [ ] none

Calibration statistics - report calibration statistics (for example, Calibration-in-the-large/slope, calibration plots) and their statistical significance (for example, P-values, confidence intervals). One can also apply resampling method (for example, bootstrapping, cross-validation)

- [x] a calibration statistic and its statistical significance are reported
- [x] a resampling method technique is applied
- [ ] none

Prospective study registered in a trial database - provides the highest level of evidence supporting the clinical validity and usefulness of the radiomics biomarker

○ yes

◉ no

Validation - the validation is performed without retraining and without adaptation of the cut-off value, provides crucial information with regard to credible clinical performance

- [x] No validation
- [ ] validation is based on a dataset from the same institute
- [ ] validation is based on a dataset from another institute
- [ ] validation is based on two datasets from two distinct institutes
- [ ] the study validates a previously published signature
- [ ] validation is based on three or more datasets from distinct institutes

Comparison to 'gold standard' - assess the extent to which the model agrees with/is superior to the current 'gold standard' method (for example, TNM-staging for survival prediction). This comparison shows the added value of radiomics

◉ yes

○ no

Potential clinical utility - report on the current and potential application of the model in a clinical setting (for example, decision curve analysis).

○ yes

◉ no

Cost-effectiveness analysis - report on the cost-effectiveness of the clinical application (for example, QALYs generated)

○ yes

◉ no

Open science and data - make code and data publicly available. Open science facilitates knowledge transfer and reproducibility of the study scans are open source region of

- [ ] interest segmentations are open source the code is open sourced
- [x] radiomics features are calculated on a set of representative ROIs and the calculated features and representative ROIs are open source
- [x]
- [ ]

Total score **14** (38.89%)
